# Current state and future prospects for psychosomatic medicine in Japan

**DOI:** 10.1186/s13030-017-0088-6

**Published:** 2017-01-16

**Authors:** Masato Murakami, Yoshihide Nakai

**Affiliations:** 1 Sanno Hospital, International College of Health and Welfare, 8-10-16 Akasaka Minato-Ku, Tokyo, 107-0052 Japan; 2 Kansai Medical College, 2-3-1 Shinmachi, Hirakata, Osaka 573-1191 Japan

**Keywords:** Psychosomatic medicine, Holistic medicine, Primary care, Japanese society of psychosomatic medicine (JSPM), Japanese society of psychosomatic internal medicine (JSPIM), BioPsychoSocial medicine (BPSM), Yujiro Ikemi, International college of psychosomatic medicine (ICPM), Asian college of psychosomatic medicine (ACPM)

## Abstract

In this article, we describe the history and current state of psychosomatic medicine (PSM) in Japan and propose measures that could be considered based on our view of the future prospects of PSM in Japan. The Japanese Society of PSM (JSPM) was established in 1959, and the first Department of Psychosomatic Internal Medicine in Japan was established at Kyushu University In 1963. PSM in Japan has shown a prominent, unique development, with 3,300 members (as of March 2016), comprised of 71.6% of medical doctors including psychosomatic internal medicine (PIM) specialists, general internists, psychiatrists, pediatricians, obstetricians and gynecologists, dentists, dermatologists, and others. Most of the non-physician members include psychology and nursing staff specialists.

The Japanese Society of Psychosomatic Internal Medicine (JSPIM), founded in 1996, is another major society with more than 1,200 physicians that is mainly composed of internists. The first joint congress of the five major PSM societies from each field was held in 2009. They included the Japanese Society of Psychosomatic Medicine, Psychosomatic Obstetrics and Gynecology, Psychosomatic Pediatric Medicine, Psychosomatic Dental Medicine, and Psychosomatic Internal Medicine. Several subdivided societies in related medical fields have also been established for cardiovascular, digestive, dermatological, and oriental medicine and for eating disorders, pain, fibromyalgia, stress science, behavioral medicine, and psycho-oncology. JSPM and JSPIM participate in international activities including publishing *BioPsychoSocial Medicine* (BPSM) and the establishment of a sister society relationship with the Germany College of PSM. PSM in Japan has adopted a variety of professional psychotherapies, including transactional analysis, autogenic therapy, and cognitive behavioral therapy. Mutual interrelationship has been promoted by the Japanese Union of Associations for Psycho-medical Therapy (UPM).

Although PSM in Japan is functioning at a high level, there remain areas that could be improved. Among the 81 medical schools in Japan, just eight university hospitals have an independent department of PSM and of 29 dental schools only three dental university hospitals have a department of psychosomatic dentistry.

Further accumulation of evidence regarding the mind-body relationship in clinical and basic science that is based on the latest advanced technology is necessary. The psychosomatic medicine community needs to make an even greater contribution to meeting the needs of modern society. The possibilities for the future development of PSM in Japan must be widely discussed.

## Background

Modern medicine developed based on the concept of the independence of the body and mind, and current clinical medicine has been subdivided into individual organ based medical practice. However, the clinical course and prognosis of ordinary physical diseases in the various clinical fields is deeply related to psychological, social, behavioral, and environmental factors, including activities of daily living. Whereas in modern medicine in which specialized psychiatrists aim to treat specific mental disorders, psychosomatic medicine (PSM) seeks “unification of mind and body” with a focus on the mind-body connection (psychosomatic relationship) [1].

PSM, which originated in Germany before spreading to the United States, was introduced into Japan after World War II by Profs. Yujiro Ikemi (1914–1999) and Shigeaki Hinohara (1911-). PSM in Japan developed as both a critique of and reflection on the prevailing animal-based research of the time, along with what was viewed as a dehumanized society resulting from industrialization and Westernization. Since its development, PSM has contributed to the development of education and research into both holistic and clinical medicine.

The mission of PSM is to become the backbone of medicine and medical care based on a foundation of holistic medicine. In this sense, PSM should be a cornerstone of clinical medicine. However, numerous contradictions and discrepancies exist between the ideal aims and the reality of PSM in Japan. Here we discuss potential causes of stagnation and future measures that can be taken to create a clear role for PSM in Japan.

## Main text

### Current organizational structures of PSM in Japan

#### Foundational aspects of PSM

In 1963, Prof. Ikemi founded the Department of Psychosomatic Internal Medicine at Kyushu University, the first medical department in Japan to specialize in PSM, and was the founder and first president of the Japanese Society of Psychosomatic Medicine (JSPM). He proposed and developed the “bio-psycho-socio-eco-ethical model,” with ecological referring to nature, including the environment, and with ethical referring to spirituality. This allowed PSM to be integrated with internal medicine and made psychotherapy more widely available [2, 3].

**Fig. 1:**
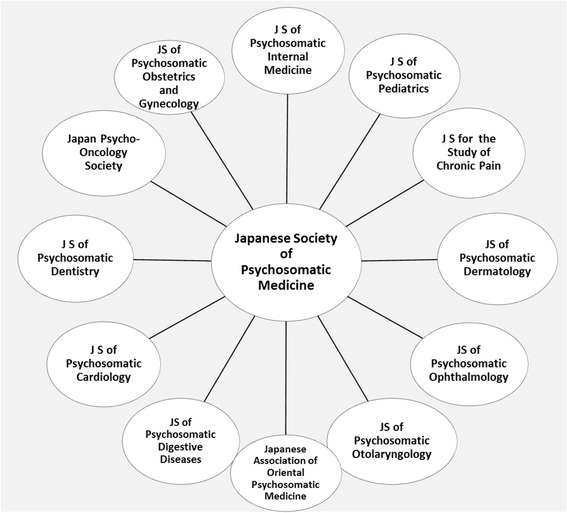
Interdisciplinary nature of psychosomatic medicine in Japan. PSM in Japan has been integrated with several areas of clinical medicine to expand its approach. The concept of PSM has been applied to and spread throughout interdisciplinary areas

PSM in Japan has aimed at being a fundamental core of holistic medicine that integrates clinical practice, education, and research in numerous fields, including adolescent medicine, primary care, family care, palliative care, home health care, geriatric care, rehabilitation, pain relief, occupational stress, and the prevention of lifestyle-related diseases. At the same time, as a specialty, PSM has been integrated with several areas of clinical medicine to expand its approach. The concept of PSM has been applied to and spread throughout interdisciplinary areas such as medical education, teamwork medicine, primary care, palliative care, psycho-oncology, chronic pain, behavioral medicine and interdisciplinary medicine. Currently, PSM specialists are expanding into various medical fields (Fig. 1), which suggests that PSM still has much potential for the future.

#### Establishment of the JSPM and the expansion of PSM into other clinical fields

The JSPM, which was founded in 1959, celebrated its 50th Anniversary in 2009. It more than doubled in size from 1,794 members in 1985 to 3,633 members in 2004; however, as of March 2015, membership had declined to 3,300.

These 3,300 members comprised 2,361 (71.6%) medical doctors, including 708 (30%) internists, 647 (27.4%) psychiatrists, 484 (20.5%) specialists of psychosomatic internal medicine (PIM), 119 (5%) pediatricians, 67 (2.9%) obstetricians and gynecologists, 65 (2.8%) dentists, 40 (1.7%) dermatologists, and 231 (9.8%) others. Psychology (medical psychologists, clinical psychologists, industrial counselors, etc.) and nursing staff accounted for nearly half of the 939 non-physician members [4]. This varied membership highlights the interdisciplinary characteristics of the JSPM (Table 1).

**Table 1:** Membership of the Japanese Society of Psychosomatic Medicine (as of March 2015)

Internists	708 (30%)
Psychiatrists	647 (27.4%)
Specialists of psychosomatic internal medicine	484 (20.5%)
Pediatricians	119 (5%)
Obstetricians and gynecologists	67 (2.9%)
Dentists	65 (2.8%)
Dermatologists	40 (1.7%)
Other	231 (9.8%)
Total physicians	2,361 (71.6%)
Total non-physician members psychology staff (medical psychologists, clinical psychologists, industrial counsellor, etc.) and nursing staff account for nearly half	939 (28.4%)
Total members	3,300 (100%)

**Fig. 2:**
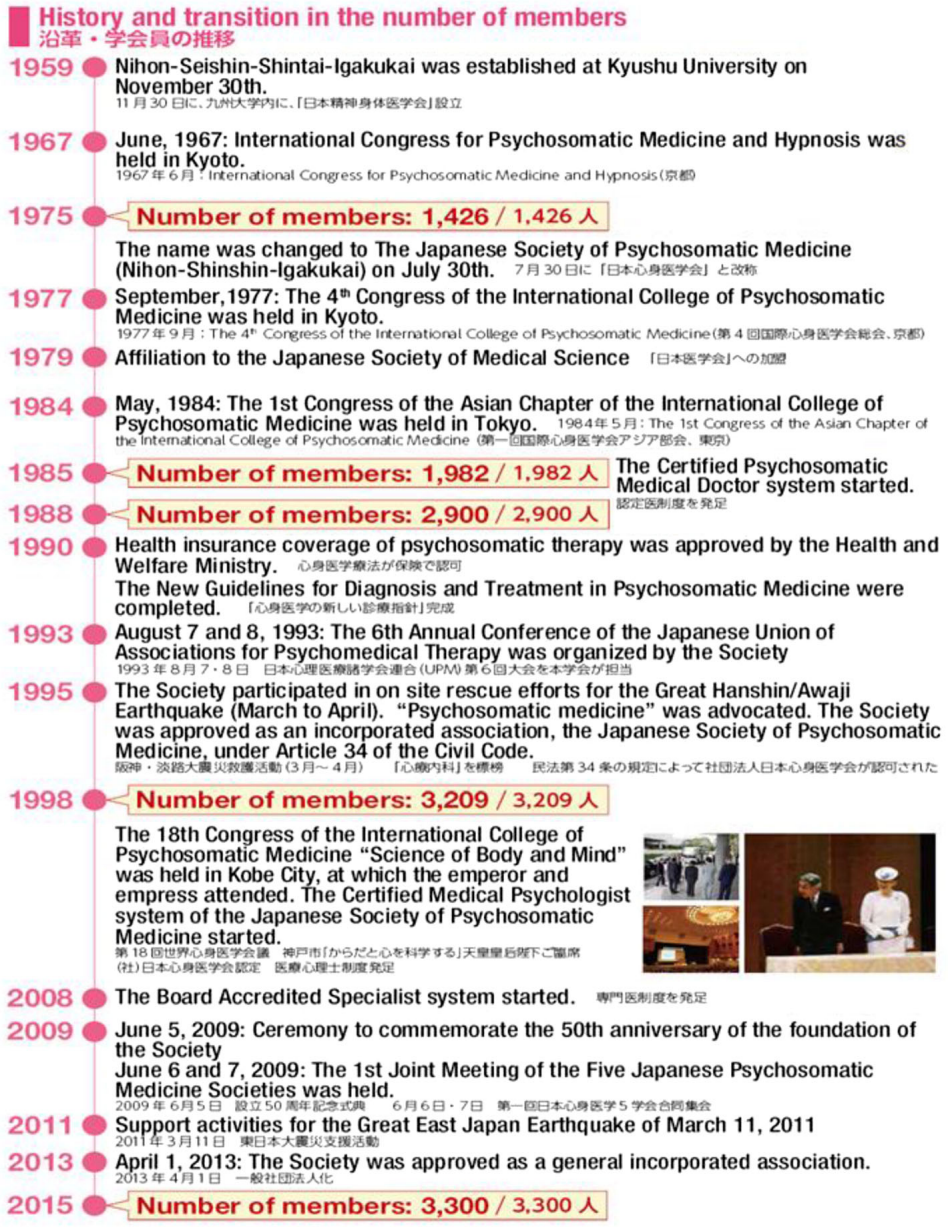
Major historical events and activities of the Japanese Society of Psychosomatic Medicine (JSPM) over the past 55 years. The JSPM was founded in 1959 and it more than doubled in size, from 1,426 members in 1975 to 3,300 members in 2015. In 1977, the 4th International College of Psychosomatic Medicine (ICPM) was held in Kyoto for the first time in Japan, chaired by Prof. Ikemi. 28 years later, the 18th ICPM was held in Kobe in 2005, with 1,100 participants from 30 countries. The Emperor and Empress of Japan attended the open ceremony and reception

The major historical events and membership of JSPM over the past 55 years are shown in Fig. 2.

A major benefit of an interdisciplinary society is the availability of relationships with a variety of specialists. However, the JSPM is mostly made up of internists, psychiatrists, and clinical psychologists. The reason for this phenomenon is the establishment of numerous specialty societies in various PSM fields, including PSM obstetrics and gynecology, PSM pediatric medicine, PSM dental medicine, PSM cardiovascular medicine, and PSM digestive medicine. Furthermore, the importance of PSM has been recognized in a number of other related societies in Japan, including those for stress science, palliative medicine, primary care, suicide prevention, pain disorders, eating disorders, fibromyalgia, and oriental medicine. This degree of interdisciplinary recognition has not been observed in other countries. However, more than half of the members of these societies do not belong to either the JSPM or the Japanese Society of Psychosomatic Internal Medicine (JSPIM). Therefore, measures to improve the situation are needed in consideration of the future of both the JSPM and the JSPIM.

The primary disadvantage of the JSPM is the difficulty in establishing a unique identity. From the beginning, the JSPM has developed through collaboration between internists and psychiatrists. Internists have learned about the diagnosis and treatment of anxiety and depressive disorders, as well as the psychoanalytic approach. Psychiatrists have learned about physical medicine and the mind-body connection. Many psychiatrists who specialize in liaison and general hospital psychiatry used to be core members of the JSPM. It was the partnership and collaboration between the two that became the driving force behind the development of the JSPM.

In 1996, PIM was authorized as a subspecialty in the field of internal medicine by the Ministry of Health and Welfare. The JSPIM was founded in 1997 by internists who held concerns about the future of the JSPM. Since the founding of the JSPIM, internists and psychiatrists have gradually transformed from being cooperative to competitive or even conflictive. In many cases, competition promotes development, but in this case, holding different identities within an interdisciplinary society may have the opposite effect. Further cooperative efforts may be needed to delineate the differences between these specialties.

#### Establishment of the JSPIM

The JSPIM was founded in 1996 by key member internists from the JSPM, therefore, many of the leading members of the JSPM are also leading members of the JSPIM. As of October 2016, the JSPIM had 1,292 members, with 88% physicians, particularly in primary care.

Most JSPIM members range in age between 40 and 50 years, and recently, a number of young physicians have expressed their intention to join. Many physicians may have joined the JSPIM because they felt that their skills in treating physical illness were limited without PSM.

The JSPIM was the first Japanese medical society to be certified as a nonprofit organization (NPO). One of the reasons for this approval was because the JSPM made substantial contributions to relief efforts in disaster-stricken areas after the Great East Japan earthquake. The JSPIM dispatched a volunteer disaster assistance team and solicited donations in collaboration with the Iwate Medical Association. This activity has been in progress for five years and continues to receive support by generous donations from outside members.

NPOs are primarily supported by people who are interested in charity and social enterprise. In addition to helping a cause they believe in, contributors receive the added benefit of tax deductions for their donations. One issue that should be addressed by the JSPIM is the public promotion of its social activities. The JSPIM celebrated its 20th anniversary in 2015 [5]. A brief history of the JSPIM’s major social activities is shown in Table 2.

**Table 2:** Major activities of the Japanese Society of Psychosomatic Internal Medicine (JSPIM) over the past 20 years

1) Certification system for registered PIM physicians
2) Certification system for PIM specialists
3) The First Commemorative Joint Congress of five Psychosomatic Medicine Societies
4) Authorization of “PIM specialist” license by Ministry of Health, Labor and Welfare
5) Certified NPO Accreditation by the Governor of Chiba Prefecture
6) Sister Society Agreement between JSPIM and DKPM (Deutsches Kollegium für Psychosomatische Medizin)
7) Inauguration of the Katsura Memorial Conference on the Therapeutic Self and the Committee on self-evaluation of the therapeutic self.
8) Translation and publication of “Therapeutic Self” authored by Watkins
9) Dispatch of a disaster assistance team to the disaster stricken area of the East Japan earthquake.
10) Dispatch of physicians to Rikuzen-takata clinic (in quake-hit area) in cooperation with Iwate Prefecture Medical Association (from October 2011 to present)
11) Collaboration with the Japan Primary Care Association
12) Membership: 1,292; physicians 87.7%, non-physicians 12.3% (as of October 2016)
13) 441Registered PIM physicians and 122 PIM specialists. (as of May 2016)

#### PSM in medical education

Among the 81 medical schools in Japan, only six (Kyushu University, University of Tokyo, Kagoshima University, Kansai Medical University, Kinki University, Toho University) have an independent department of PSM. Two other universities have PSM divisions only in their hospitals (Tohoku University, Nihon University) Among the 29 dental schools in Japan, Tokyo Medical and Dental College is the only one that has an independent Department of Psychosomatic Dentistry. Nihon University and Nihon Dental College are the only two institutions that have PSM divisions in their dental hospitals (annotation: A Department of PSM is to be established at the newly founded International University of Health and Welfare Medical School in 2017).

Due to a shortage of members, the contributions of PSM to medical education remain limited. Only a few specialists are active in university hospitals and general medical facilities. Under such circumstances, it might be considered a minor miracle that the JSPM has been able to maintain 3,300 members.

#### Differences and similarities between psychiatry and PSM

PSM in Japan has played a founding role and maintained its characteristic interdisciplinary features and generality. In cooperation with psychiatrists, physicians such as internists, gynecologists, and pediatricians in Japan have participated in the realization of the ideal PSM.

In 1992, the JSPM defined the concept of psychosomatic diseases (PSDs) as follows: “Psychosomatic diseases are pathological dysfunctions, including organic and functional physical disorders, in which psychosocial factors are closely involved in the onset and course, excluding physical symptoms associated with other mental disorders, such as neurosis and depression.”

We asked the late Prof. Ikemi how this definition was established. He noted that the intention behind the definition was to differentiate the professional from the psychiatric position. This concept of PSD has been widely accepted by Japanese physicians, including most of the psychiatrists, through the remarkable efforts of Prof. Ikemi. However, the structure of disease has been diversifying due to drastic changes in the social structure and environment, compared with the situation in Prof. Ikemi’s time, and this differentiation has become increasingly difficult. This problem is not limited to only PSM; it affects every area of medicine because the boundaries between specialties have become blurred and an increase in the number of unsolvable cases has been observed, regardless of the technical knowledge or skills in the various medical fields.

Most patients with depression, an anxiety disorder, or a somatoform disorder initially visit a primary care physician or a non-psychiatrist. The symptoms of many mental disorders are comorbid with PSDs, and thus distinguishing between them is difficult.

If it is necessary to reestablish the definition of PSDs, this comorbidity must be taken into consideration. An honest and frank exchange of views regarding the mind-body relationship is needed between physicians and psychiatrists, as an ambiguous stance on such issues may cause confusion and adversely affect social understanding.

#### Public misconceptions regarding PSM

Many people in Japan have prejudices and misunderstandings about psychiatry and PSM. This should not be surprising because many physicians also have misperceptions. According to a previous investigation, the proportion of psychiatrists who advocated PIM in name only rapidly increased to more than 80%. One of the reasons is that public stigma still exists toward seeing a psychiatrist, and psychiatrists tend to choose to be referred to as PIM physicians. But most of these psychiatrists do not belong to the JSPM or the JSPIM. Because physicians can freely advocate any specialty under Japanese medical law, many people hold the misconception that PSM is the same as psychiatry.

It is regrettable that proper education regarding PSM cannot be provided in medical school or during residency because only about 10% of medical schools in Japan have well-organized PSM departments. Many people therefore misunderstand PSM as a field of psychiatry that deals with psychiatric diseases. Although the members of the JSPM may be responsible for this situation, we must put forth our best efforts to promote the society and clear up such misunderstandings.

#### Medical expenses in PSM

Although it takes substantial time and effort to care for patients with intractable PSM diseases, the amount of medical expenses covered by public health insurance for PSM treatment in Japan is extremely low. As of November 2016, PSM treatment typically costs 1,100 JPY (about 10 USD) for the first visit and 800 JPY for repeat visits, which is considerably lower than that for psychiatric outpatients (3,300 JPY and over, over 30 USD). The cost of conventional psychoanalysis, a limited high-cost psychotherapy that PSM specialists are eligible to conduct, is about 3,900 JPY (35 USD) for a 45-min session. We actively approached the Ministry of Health, Labor and Welfare concerning raising the amount they reimburse providers of PSM treatment, but this effort was in vain. However, some progress should be noted in that an increase was approved for the treatment of inpatients with eating disorders.

Under these circumstances, young doctors who aspire to having a career in PSM may lose motivation. In addition, the number of medical students who wish to become PSM physicians is decreasing. Increasing medical expenses may be the key to encourage an increase in the membership of the JSPM.

#### Psychotherapy in PSM in Japan

It is noteworthy that PSM in Japan has adopted a variety of professional psychotherapies. PSM specialists have contributed to the development of the Japanese Society of Transactional Analysis (JSTA) [6], the Japanese Society of Autogenic Therapy (JSAT) [7], and the Japanese Association of Behavioral Cognitive Therapies (JABCT), which Prof. Ikemi had designated as the three major pillars for PSM specialists.

The Japanese Union of Associations for Psycho-medical Therapy (UPM) was established in 1987. As of 2016, 15 medical societies, such as the JSPM and JSPIM, and psychological societies, such as the JSTA and JABCT, are members of the UPM, whose participants are able to learn about combined medicine and psychology.

### International cooperation of Japanese PSM

#### Contributions to the international congress of psychosomatic medicine

From August 21 to 26, 2005, the 18th International College of Psychosomatic Medicine (ICPM) was held in Kobe, Japan. It was chaired by Profs. Chiharu Kubo (Kyushu University) and Tomifusa Kuboki (Tokyo University). It was a notable event because the 4th ICPM had been held for the first time in Japan (chaired by Prof. Ikemi) in Kyoto 28 years previously, in 1977. The Emperor and Empress of Japan attended the open ceremony and reception. It was a huge success, with more than 1,100 participants from 30 countries.

It was extremely meaningful that the ICPM was held in Kobe 10 years after the Great Hanshin-Awaji earthquake. Many JSPM members were affected by the disaster, and many were mobilized as volunteers. The theme of the congress was “Let’s Get Together in Japan, a Country with a Long Tradition of Mind-Body Awareness, to Discuss Scientific Approaches to the Mind and Body!” We believe we were able to make great strides in communicating the strengths of Japanese PSM to the participants from other countries. During the period of 2010–2014, Prof. Kubo demonstrated excellent leadership as the president of the ICPM (Fig. 2).

The Secretariat of the Asian College of Psychosomatic Medicine (ACPM) is located in the Department of PSM at Kyushu University. In 2016, Prof. Kubo, President of Kyushu University, took the position of Chief Director of the ACPM to contribute to the development of PSM in other Asian countries, including Korea, China, Taiwan, India, Indonesia and Mongolia.

#### International PSM journal

In January 2007, the JSPM began publishing their official international journal, *BioPsychoSocial Medicine(BPSM)*. The submission of numerous articles from abroad and the journal’s international readership has been of great importance to the introduction, promotion, and development of Japanese PSM around the world.

Recently, a decrease has been observed in the number of submissions of original articles in Japanese to the official Japanese journal *Shinshin-igaku*. It may therefore be necessary to shift the priority from the domestic journal to the international journal in the near future.

#### Sister society relationship between the JSPIM and the DKPM

**Fig. 3:**
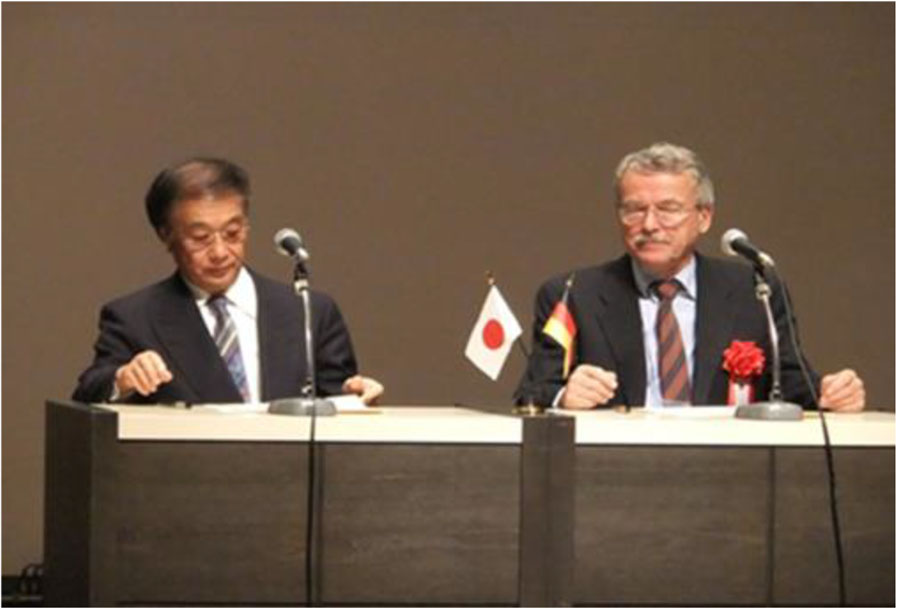
Signing ceremony for the establishment of a sister society relationship between the JSPIM and the DKPM. A signing ceremony for the establishment of a sister society relationship between the JSPIM and the Deutsches Kollegium für Psychosomatische Medizin (DKPM) was held at the 16th Congress of the JSPIM on November 26–27, 2011 (Chaired by Prof. Masato Murakami, Nihon University). Shown in the photo are Profs. Yoshihide Nakai (President of the JSPIM) and Hans Christian Deter (President of the DKPM)

In 2011, which also marked the memorable occasion of the 150th Anniversary of the Japan-Germany Trading Exchange, a sister society relationship between the JSPIM and the *Deutsches Kollegium für Psychosomatische Medizin* (DKPM) was established at the 16th Congress of the JSPIM (Chaired by Prof. Masato Murakami, Nihon University). Profs. Yoshihide Nakai (President of the JSPIM) and Hans Christian Deter (President of the DKPM) attended the signing ceremony [8] (Fig. 3). Since that time, both societies have maintained a close relationship through interaction at annual meetings and international congresses.

PSM is seeing remarkable growth in both Germany and Japan, and the following similarities can be observed: 1) PSM was developed by non-psychiatrist physicians; 2) both countries have independent PSM departments in medical schools and university hospitals; 3) nearly the same diseases are targeted; and 4) PSM has a history of both cooperation and conflict with psychiatry.

The Japanese term “*shinryo-naika*” translates as “psychosomatic internal medicine.” The literal meaning of “*shinryo*” is psychotherapy, and “*naika*” is internal medicine. Therefore, “*shinryo-naika*” literally means “internal medicine specializing in psychotherapy.” “*Shinryo-naika*” in Japan is comparable with the Department of Psychosomatic Medicine and Psychotherapy (*Psychosomatische Medizin und Psychotherapie*) in Germany.

In Germany, almost all of the medical universities or university hospitals have established a Department of Psychosomatic Medicine and Psychotherapy, and knowledge of PSM is indispensable for the National Examination of Medicine. Furthermore, PSM training is essential in medical school and during residency.

As of 2011, there were 4,637 PSM specialists in Germany, with 120 new specialists entering the field annually from 2001 to 2011. For more information you can see “*Psychosomatische Medizin und Psychotherapie Heute*” (eds. Herzog, Beutel, and Kruse) or the Japanese translation by Makoto Hashizume MD, which was published in 2015 [9].

Both the JSPIM and the DKPM consider worldwide promotion of the necessity of PSM and a new medical model to be an important mission. The fusion of Eastern and Western PSM wisdom might lead to profound insights, thus further development of mutual interaction between these two spheres is expected.

#### Worldwide and domestic PSM

Those in the field of PSM in Japan are proud of its worldwide scale and aim for its increased promotion by overcoming language barriers. Our official international journal, *BioPsychoSocial Medicine*, will play an important role in this process. By utilizing more active international communication via joint academic meetings and through personnel exchanges, we hope to transform domestic PSM into worldwide PSM.

### Specialization in Japanese PSM

#### The specialty board system in PSM

The JSPM specialty board system was established in 1985 and, as of August 2014, had registered 581 PSM specialists. The field of specialization in PSM is denoted in parentheses after the name of the PSM specialist. For example, “Name of PSM specialist (Internal Medicine)”; the same goes for (Psychiatry), (Pediatrics), (Obstetrics and Gynecology), etc. In order to be licensed as a PSM specialist (Internal Medicine), applicants need to be qualified by the specialty board in the relevant field.

The JSPIM specialty board was established in 2007 and, as of May 2016, had registered 441 PIM physicians and 122 PIM specialists. The establishment of a new specialty board system is currently in progress under the auspices of the Japan Specialist Accreditation System, which is conducted by the Ministry of Health, Labor and Welfare. PMS specialists (internal medicine) and PIM specialists may also be included as a subspecialty in a core society, such as the Japanese Society of Internal Medicine.

#### Medical psychologist system

In 2004, the JSPM specialty board of certified medical psychologists was established. Implementation guidelines were devised and qualifying examinations were conducted. As of June 2016, a total of 82 medical psychologists had been certified. PSM in Japan has developed in collaboration with co-medical staff, and thus training facilities will need to be established to provide a model for teamwork medicine.

### The future of PSM in Japan

This brings us to the question of what will the state of PSM in Japan be in 50 years? It is certain that medicine and medical care will become even more highly advanced with the use of cutting-edge technology, such as computerized models, regenerative medicine, gene therapy, and robotic surgery. In the future, medical care will likely place an emphasis on rationality, efficiency, functionality, and other performance-based principles. In contrast to this trajectory, many patients will still require handmade medicines and more humanistic medical care.

Therefore, the development and contributions of PSM required in modern society will need to be thoroughly investigated over time. Research on stress science and coping from a global perspective will be needed in consideration of the current social environment. PSM is expected to become increasingly important in the future.

#### The mission of PSM

Ideally, PSM should be practiced across all medical areas as an interdisciplinary field of study. The main principle of PSM is based on relational system theory and the bio-psycho-social medical model. PSM in Japan is based on a clear mission to emphasize the role of multiple factors in disease formation. The future of medicine is moving in the direction of more advanced technology. To earn the trust of those receiving medical services, it will be important to effectively utilize advanced technology in clinical practice and to establish education and research as pillars of holistic medicine. However, this model remains just theory and concepts; only few evidence-based studies have been performed, and it remains unclear how it could be utilized in clinical practice, education, and research. We should further investigate this concept for the future of PSM in Japan.

The foundations of a building are not easily visible, and visible foundations can lose their meaning. However, even the best-designed building cannot be built without a foundation. The process of laying down groundwork is the mission of Japanese PSM.

#### Development of research methodology

PSM is a relational medicine based on system theory, and thus research based on a traditional linear model that focuses on individual factors may result in contradictions. The necessity of new research methods based on a non-linear model has been emphasized. The accumulation of knowledge in PSM will contribute to the elucidation of the pathogenesis and mechanisms of many stress-related phenomena and disorders such as chronic pain, chronic fatigue, medically unexplained physical symptoms, and functional somatic symptoms.

#### Research on the mind-body relationship

Research on the mind-body connection is one of the most important missions of PSM. Using the latest advanced technology, fundamental research in PSM has become limitless in fields involving DNA sequencing and gene expression as modulated by environmental factors, the analysis of protein-metabolite proteomes and metabolomics, immune modulation by oral bacterial flora in the gut and brain, neuroimaging studies on neural activity, and the discovery of biological markers. The further accumulation of evidence regarding the mind-body relationship in clinical and basic science is necessary. Because PSM is also somatopsycho, so-called mind-body unity should be kept in mind.

#### Research on the economic effects of PSM

It is also important to study the economic effects of PSM in terms of medical expenses and increased patient satisfaction. For example, it is worthwhile to study the economic effects of the application of PSM for the treatment of diabetes and hypertension. To investigate this issue, it will be important to establish a research team made up of members of the society who can publish their results and present their data at international meetings.

#### PSM for the elderly

PSM for the elderly has yet to be adequately explored. Aging societies are becoming a major issue, not only in Japan but also in many other countries around the world. Geriatric medicine is one of the fields for which PSM is expected to be able to make a substantial contribution.

Japan is the most aged society in the world. As of 2014, the average life expectancy in Japan was the highest in the world (80.5 years for men, 86.3 years for women). In addition, the percentage of the population over the age of 65 years was in excess of 26% in 2014 and is expected to be over 41% by 2050. The current methods of providing support to the elderly are now being called into question. The late Prof. Jin-ichi Suzuki (Tohoku University) used to emphasize the necessity of body-oriented somato-psychotic medicine in geriatric PSM.

Medical care for the elderly requires vast networking between the primary care physician, family medicine physicians, public health nurses, nurses, caregivers, hospitals, regional communities, health centers, and community health care teams. PSM specialists are the most appropriate professionals to perform teamwork medicine in collaboration with co-medical staff and primary care physicians.

#### Application of PSM in primary care

PSM and primary care medicine have maintained a close relationship. In 1978, the late Prof. Suzuki, as a board member of the Japan Primary Care Association (JPCA), was thoroughly engaged in the establishment of this relationship. Prof. Michio Hongo (Tohoku University) has inherited this role. In addition, the late Prof. Ikemi chaired the 5th Congress of the JPCA in 1982, where the main theme was “The practice of holistic medicine in primary care.”

Michael Balint emphasized the need to “cultivate intrapersonal communication between patients and physicians for good treatment from the biopsychosocial point of view.” He also stated that, compared with psychiatrists, general practitioners with PSM skills would be able to provide more appropriate therapy to patients with common diseases. Systematic collaboration between primary care medicine and PSM is expected to become more important in the near future.

#### Importance of joint meetings of PSM related societies

**Fig. 4:**
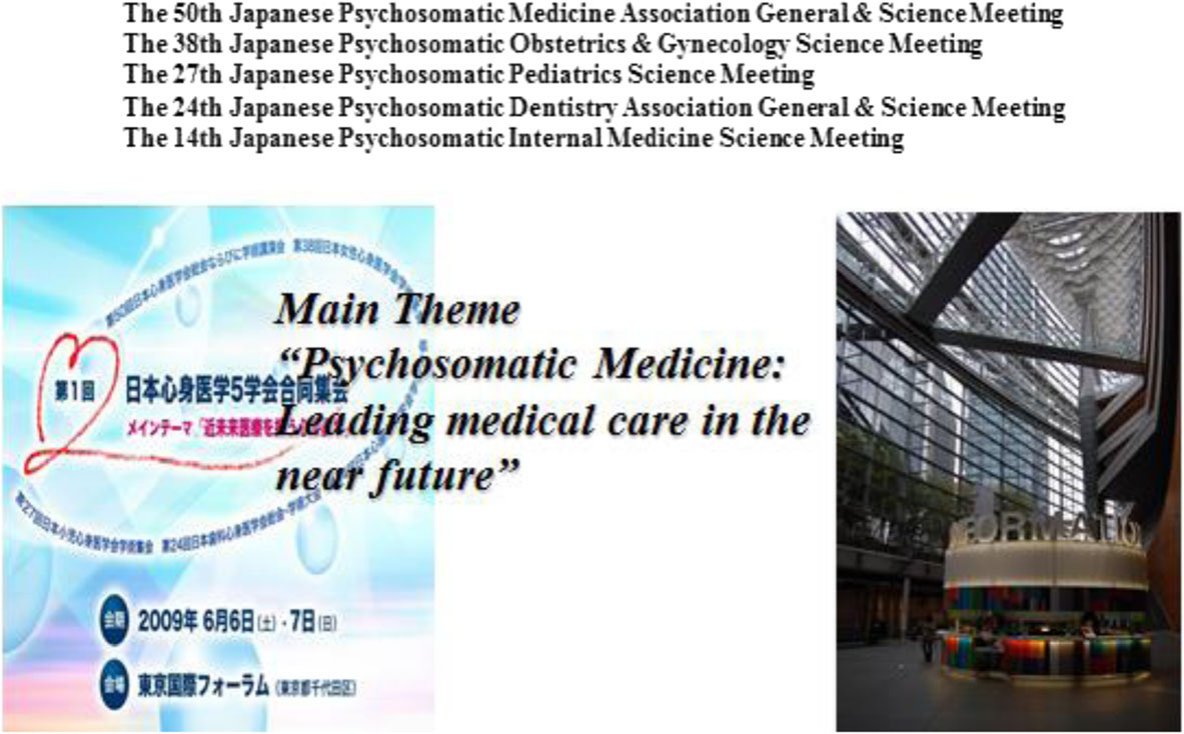
The 1st joint congress of the five major PSM societies in Japan. The 1st joint congress of five major PSM societies was held on June 6–7, 2009 at the Tokyo International Forum. The total number of members of all five societies was 7,092. The main theme was “The important role of psychosomatic medicine in modern medical care in the near future”

As previously mentioned, there are five major PSM societies in Japan. The 1st joint congress of these five societies was held on June 6–7, 2009 at the Tokyo International Forum (Fig. 4). The participating societies were as follows (in order of establishment): the Japanese Society of Psychosomatic Medicine (JSPM; 1959, 3,776 members at that time); the Japanese Society of Psychosomatic Obstetrics and Gynecology (JSPOG; 1972, 671 members); the Japanese Society of Psychosomatic Pediatric Medicine (JSPPM; 1983, 800 members); the Japanese Society of Psychosomatic Dental Medicine (JSPDM; 1985, 565 members); and the Japanese Society of Psychosomatic Internal Medicine (JSPIM; 1996, 1,280 members).

Very little member overlap was observed between the five societies. The total number of members of all five societies was 7,092. The main theme was “The important role of psychosomatic medicine in modern medical care in the near future,” and the subthemes of each society included “The lifecycle and psychosomatic medicine” (JSPOG), “The carryover of children’s psychosomatic diseases” (JSPPM), and “Aim to be a good clinician” (JSPIM).

To further develop PSM throughout Japan, it is desirable to organize joint congresses between these five related societies with the common theme of deepening and promoting relationships and interaction. Examples of cross-sectional themes could include “Lifestyle-related diseases and PSM”, “Holistic medicine in clinical medicine”, and “Gender-based medicine and PSM”. Innovative prospects can be expected only through the participation of these five societies.

#### Construction of networks and enlightening activities

Recently, various symposiums and meetings have been organized by young PSM physicians, especially in the field of respiratory medicine, which should be highly welcomed. These young physicians are expected to provide unique ideas for enlightening activities and the construction of networks. Opportunities for success should be provided for young physicians by the board of trustees of both the JSPM and the JSPIM.

## Conclusions

Japan is located at the crossroads, both geographically and culturally, of East and West. Therefore, our mission is to become a bridge between Eastern and Western PSM. Both the JSPM and the DKPM have been actively exchanging information since reaching an affiliation agreement. Society is undergoing transformation increasingly rapidly, and a variety of issues that are not covered by traditional concepts and models of PSM have been revealed. State-of-the-art medicines and technologies, such as robotic surgery, are expected to be the primary issues of medical care in the near future. Humanistic medicine is indispensable in opposition to these changes. Bringing PSM to the frontiers of medicine can be promoted through international cooperation between members of worldwide societies.

## Abbreviations

ACPM: Asian college of psychosomatic medicine
BPSM: BioPsychoSocial Medicine
DKPM: Deutsches kollegium für psychosomatische medizin
DKPM: Deutsches kollegium für psychosomatische medizin
ICPM: International college of psychosomatic medicine
JABCT: Japanese association of behavioral cognitive therapies
JPCA: Japan primary care association
JSAT: Japanese society of autogenic therapy
JSPDM: Japanese society of psychosomatic dental medicine
JSPIM: Japanese society of psychosomatic internal medicine
JSPIM: Japanese society psychosomatic internal medicine
JSPM: Japanese society of psychosomatic medicine
JSPOG: Japanese society of psychosomatic obstetrics and gynecology
JSPPM: Japanese society of psychosomatic pediatric medicine
JSTA: Japanese society of transactional analysis
PIM: Psychosomatic internal medicine
PSDs: Psychosomatic diseases
PSM: Psychosomatic medicine
UPM: Japanese union of associations for psycho-medical therapy
